# Sharing Health Data for Better Outcomes on PatientsLikeMe

**DOI:** 10.2196/jmir.1549

**Published:** 2010-06-14

**Authors:** Paul Wicks, Michael Massagli, Jeana Frost, Catherine Brownstein, Sally Okun, Timothy Vaughan, Richard Bradley, James Heywood

**Affiliations:** ^1^PatientsLikeMe Inc.Research & DevelopmentCambridgeUnited States

**Keywords:** Personal health records, data visualization, personal monitoring, technology, health care, self-help devices, personal tracking, social support, online support group, online health community

## Abstract

**Background:**

PatientsLikeMe is an online quantitative personal research platform for patients with life-changing illnesses to share their experience using patient-reported outcomes, find other patients like them matched on demographic and clinical characteristics, and learn from the aggregated data reports of others to improve their outcomes. The goal of the website is to help patients answer the question: “Given my status, what is the best outcome I can hope to achieve, and how do I get there?”

**Objective:**

Using a cross-sectional online survey, we sought to describe the potential benefits of PatientsLikeMe in terms of treatment decisions, symptom management, clinical management, and outcomes.

**Methods:**

Almost 7,000 members from six PatientsLikeMe communities (amyotrophic lateral sclerosis [ALS], Multiple Sclerosis [MS], Parkinson’s Disease, human immunodeficiency virus [HIV], fibromyalgia, and mood disorders) were sent a survey invitation using an internal survey tool (PatientsLikeMe Lens).

**Results:**

Complete responses were received from 1323 participants (19% of invited members). Between-group demographics varied according to disease community. Users perceived the greatest benefit in learning about a symptom they had experienced; 72% (952 of 1323) rated the site “moderately” or “very helpful.” Patients also found the site helpful for understanding the side effects of their treatments (n = 757, 57%). Nearly half of patients (n = 559, 42%) agreed that the site had helped them find another patient who had helped them understand what it was like to take a specific treatment for their condition. More patients found the site helpful with decisions to start a medication (n = 496, 37%) than to change a medication (n = 359, 27%), change a dosage (n = 336, 25%), or stop a medication (n = 290, 22%). Almost all participants (n = 1,249, 94%) were diagnosed when they joined the site. Most (n = 824, 62%) experienced no change in their confidence in that diagnosis or had an increased level of confidence (n = 456, 34%). Use of the site was associated with increasing levels of comfort in sharing personal health information among those who had initially been uncomfortable. Overall, 12% of patients (n = 151 of 1320) changed their physician as a result of using the site; this figure was doubled in patients with fibromyalgia (21%, n = 33 of 150). Patients reported community-specific benefits: 41% of HIV patients (n = 72 of 177) agreed they had reduced risky behaviors and 22% of mood disorders patients (n = 31 of 141) agreed they needed less inpatient care as a result of using the site. Analysis of the Web access logs showed that participants who used more features of the site (eg, posted in the online forum) perceived greater benefit.

**Conclusions:**

We have established that members of the community reported a range of benefits, and that these may be related to the extent of site use. Third party validation and longitudinal evaluation is an important next step in continuing to evaluate the potential of online data-sharing platforms.

## Introduction

Managing complex and chronic diseases is a difficult undertaking for patients and clinicians alike. Clinicians are only able to allocate an average of 15 minutes per patient visit [1], frequently use medical jargon that patients do not understand [2], and are also unable to devote enough time to collaborative decision making with their patients [3]. These factors disproportionately affect the socially disadvantaged, such as patients from racial and ethnic minority groups and those with low literacy and low socioeconomic status [4]. For their part, patients do not adhere fully to medical advice [5], miss appointments [6], and use complementary and alternative medicines with little evidence to support these methods [7], often without informing their physician [8]. Although modifying the way clinicians interact with patients can yield some benefits, there is also a corresponding benefit to be gained in educating patients about how to make better use of their health care visits through intensive training programs such as the United Kingdom’s “Expert Patient Programme” [9]. However such programs require extensive logistical support and require winning stakeholder “buy-in” from a range of agencies.

The Internet provides a platform to develop efficient, sustainable online resources for patients to research their medical questions, communicate with one another, and support each other, such that patients assume more responsibility for their care and decrease the burden on the health care system. Most American adults (74%) have access to broadband Internet connections, and 61% look online for health information [10], although there are barriers to access for those with a chronic illness [11]. The simplest method for online interaction is email, which even older patients are enthusiastic to use [12]; their physicians, however, are not [13], with estimates of only around 7% of physicians communicating with their patients in this way.

In recent years a number of online communities have been developed by patient organizations, providers, and nonprofit organizations. Such online communities are virtual forums where patients can discuss their health concerns and exchange information. Successful examples of such sites include Braintalk for neurological diseases [14] and Building User Involvement in Motor Neurone Disease (BUILD) for amyotrophic lateral sclerosis (ALS) [15]. While evidence about the impact of participation in online communities on medical outcomes is limited, the psychological benefits and increased quality of patient-physician interactions have been demonstrated [16].

Participation in online communities heightens levels of emotional well-being, perceived control over disease, overall personal empowerment, and level of medical knowledge [17,18]. The psychological outcomes have, in some cases, translated into improvements in medical decision making and positive behavioral change (see Barak et al [18] for a review). Still, concerns remain about potentially detrimental effects of online communities. Providers are concerned about patients diagnosing themselves or finding misinformation online [19]. However, these concerns appear unfounded when examined systematically [16,20], and chronic disease patients themselves are relatively well-informed about the potential limitations of the Internet as a source of information [21].

PatientsLikeMe is a Web-based application where members explicitly choose to share detailed computable data about symptoms, treatments, and health in order to learn from the experience of others and improve their outcomes. These data are presented back to members as individual-level graphical health profiles and aggregated into reports accessible on the site. Members can discuss these data sets either within a group forum or individually through private messages. The resources on the site are designed to help members answer the question: “Given my status, what is the best outcome I can hope to achieve, and how do I get there?” The platform itself has been described previously in detail [22,23,24] and is gaining recognition for its clinical research in areas as diverse as patient-reported outcomes [25], identification and quantification of symptoms in neurological diseases [26], patient education and decision-making [27,28], and patient-lead clinical trials [29]. The site fulfills Fox’s definition of a tool helping patients to find a “just-in-time, someone-like-me” peer that can be relied upon to compare options and aid decision making [10].

To date, we have not analyzed the potential psychological and health services outcomes associated with site usage. In this paper, we present an initial study of the potential benefits of sharing health data in the site. We sought to describe the potential benefits of PatientsLikeMe and to understand the effect of our novel functionality on patient-reported outcomes. Specifically, we explored the following research questions: Do members of PatientsLikeMe perceive benefits from participating in this online community? Do they think that they make better treatment decisions? Do they feel they are managing their symptoms better? Do members become more engaged in their health care decisions? Does the site influence health care and outcomes? If so, is this related to site use? Finally, what are members’ attitudes toward sharing data on the site?

## Methods

We used the PatientsLikeMe survey system (PatientsLikeMe Lens) to construct a core set of questions (Multimedia Appendix 1) which were answered by registered members of PatientsLikeMe.com in the following communities: ALS, multiple sclerosis (MS), Parkinson’s disease (PD), human immunodeficiency virus (HIV), mood disorders, and fibromyalgia. The questionnaire included a core set of questions to be answered by all participants, as well a set of community-specific questions. The survey was piloted on members of our rare disease communities (progressive supranuclear palsy, multiple system atrophy, and neuromyelitis optica) to ensure comprehension and ensure that the system functioned correctly, but the sample size was considered too small to draw reliable conclusions from the data (n = 30). Even in this small sample, the full range of responses was used and patients did not report dissatisfaction with completing the survey.

### Recruitment

Members of PatientsLikeMe find out about the site through a variety of channels: search, paid advertisements, public relations, press mentions, academic collaborations, word of mouth from patients, and provider referral. Most members (approximately 80%) are based in the United States, with the remainder distributed throughout the world, predominantly in the English-speaking world, although members in some countries use translation software to participate.

### Checklist for Reporting Results of Internet E-Surveys

The following information is provided to comply with the Checklist for Reporting Results of Internet E-Surveys [30]. A systematically selected probability sample of members from each community was invited to participate in the survey by electronic private message in March 2009. New private messages triggered an automated email to patients’ email accounts (unless they had opted out of being contacted in this way). Sampled patients had their own password-protected login, had previously submitted age and sex data, and had been members of the site for at least 30 days. Patients could only complete the survey once, and we have tools to prevent multiple accounts originating from the same location, including account registration, cookies, and IP tracing. Therefore, we have more confidence in our denominators than might be found using an “open” survey method. The survey was voluntary and completion was not required to continue using the other features of the site. No incentives were offered; question order was not randomized; certain items only appeared conditional on previous responses (ie, were “adaptive”) to minimize respondent burden (see Multimedia Appendix 1); and the total number of questions and screens varied by community and participants’ responses.

Following initial contact, a reminder message was sent within a week to those who had not yet completed the survey; patients who had only partially completed the survey could reaccess it through the original private message (or reminder message) to complete their survey. Once opened, the survey had a “back” button that allowed participants to change their earlier answers. Only data from completed questionnaires are presented here, with the exception of the analysis between site use and treatment benefit in order to maximize sample size. As an internal research project without external sponsors, and with no anticipated adverse consequences for participation, institutional review board (IRB) approval was not sought for this project. Members of PatientsLikeMe join the site with the expectation that they will be participating in research. The recruitment message (see Multimedia Appendix 1) outlined the purpose of the study, reminded patients that they were under no obligation to participate, that their aggregated results may be published, and that the survey should take about 10 minutes to complete. It was sent from user accounts for authors PW and MM, who could easily have been contacted by potential participants from within the PatientsLikeMe system.

User data was protected in accordance with PatientsLikeMe’s internal security standard operating procedures, which include password protection, deidentification of locally held data files, regular automated backup, and physical protection of IT hardware.

### Data Analysis

Data analysis was performed using Statistics Package for the Social Sciences, version 18.0 (SPSS Inc, Chicago, IL, USA). Data were assessed for normality to guide the use of parametric or nonparametric statistics. Categorical variables were assessed using chi-square; normally distributed demographic data were analyzed using Student’s *t* test or between-groups analysis of variance (ANOVA). Nonparametric between-group differences were tested using the Kruskal-Wallis test. In all cases, tests performed were two-tailed and assumed a cutoff of *P* < .05 for statistical significance.

**Table 1:** Patient characteristics and response rates by disease

Disease Community	Number of Survey Invitations Sent	Completed	Partially Completed	Opted Out	No Response	Age of Community	Median Duration of Site Use
		n (%)	n (%)	n (%)	n (%)		
MS	1992	347(17%)	14 (1%)	151 (8%)	1480 (74%)	24 months	9 months
PD	997	287 (29%)	45 (5%)	70 (7%)	595 (60%)	24 months	10 months
ALS	988	218 (22%)	41 (4%)	45 (5%)	684 (69%)	40 months	15 months
Fibromyalgia	801	150 (19%)	44 (6%)	39 (5%)	568 (71%)	5 months	2 months
HIV	1088	177 (16%)	58 (5%)	45 (4%)	808 (74%)	18 months	7 months
Mood disorders	999	144 (14%)	37(4%)	46 (5%)	772 (77%)	13 months	6 months
Total	6865	1323 (19%)	239 (4%)	396 (6%)	4907 (72%)	N/A	

**Table 2:** Demographics of respondents compared to nonrespondents by disease

	Sex			Age		
	Respondent	Nonrespondent		Respondent	Nonrespondent	
Disease Community	Number Male of Total N (% Male)	Number Male of Total N (% Male)	χ^2^ (df) *P*	Mean Age (SD)	Mean Age (SD)	*t* test, *P* (95% Confidence Interval [CI] of Age Difference)
MS	65 of 347 (19%)	312 of 1645 (19%)	0.10(1) *P* = .92	46 (9)	43 (11)	3.24, *P* = .001 (0.8 - 3 yrs)
PD	131 of 287 (46%)	404 of 710 (57%)	10.42(1) *P* = .001	60 (10)	58 (11)	2.21, *P* = .03 (0.3 - 3.2 yrs)
ALS	145 of 218 (67%)	445 of 770 (58%)	5.37(1) *P* = .02	54 (11)	53 (12)	0.13, *P* = .9 (-1.6 to 1.8 yrs)
Fibromyalgia	3 of 150 (2%)	37 of 651 (6%)	3.49(1) *P* = .06	45 (12)	45 (12)	0.64, *P* = .52 (-1.4 to 2.8 yrs)
HIV	124 of 177 (70%)	652 of 911 (72%)	0.17(1) *P* = .68	42 (11)	39 (11)	3.28, *P* = .001 (1.2 - 4.8 yrs)
Mood disorders	34 of 144 (24%)	238 of 855 (28%)	1.11(1) *P* = .29	39 (13)	37 (14)	1.9, *P* = .06 (-0.1 to 4.7 yrs)

## Results

### Participants

The overall response rate was 19% (1323 of 6825) (Table 1). There were significant differences in participation rates between communities (χ^2^_15_ = 193.78, *P* < .001), with the highest rate in the Parkinson’s disease group (n = 287, 29%) and the lowest in the mood disorders group (n = 144, 14%). Only patients who had been members of the site for more than 30 days were invited to participate; the actual duration of site usage (shown in Table 1) varied by disease; however, because the communities were themselves of differing ages, it is hard to interpret the significance, if any, of such differences.

Within disease groups there were varied patterns of significant differences between respondents and nonrespondents (including those who did not respond at all, those who opted out, and those who did not complete the survey, Table 2. MS respondents were 3 years older than MS nonresponders; PD respondents were more likely to be female and were 2 years older than PD nonresponders; ALS respondents were more likely to be male; fibromyalgia respondents were more likely to be female; HIV respondents were 3 years older than HIV nonrespondents. There were significant differences in sex ratio between diseases (c^2^_5_=309.57, *P* < .001) and in age between disease groups (F_5,1317_ = 120.06, *P* < .001). The age differences, though statistically significant are not large, and the between-condition demographic differences are to be expected given the epidemiological profile of each disease.

In the survey invitation (see Multimedia Appendix 1) we suggested that completing the survey should take approximately 10 minutes. During the development of the survey, we added an additional feature to the survey system, which allowed us to estimate the time taken to complete the survey, but this was only available for a limited subset (n = 384). For those participants, the median time to completion was approximately 12 minutes (median 743 seconds, interquartile range 560 seconds). At the end of the survey was an open text response box which stated: “Please use the space below for your final comments, or if you have a suggestion for one thing you would really like to see changed on the site please let us know here.” The full set of anonymized responses is presented in Multimedia Appendix 2.

### Treatment Decisions

The PatientsLikeMe site has been described previously [22]. Briefly, the site offers a variety of tools to help patients record the treatments they are taking, supported by a drug database to promote accurate data entry. On an individual basis, patients can see a visual display of their treatment history over time on their profiles. Data on treatment brands, dosages, duration on treatment, reasons for stopping, and evaluations of efficacy and side effects are aggregated into “treatment reports.” These tools are intended to help educate and inform patients about treatments they are using or considering.

Patients agreed with the statement that using the site had helped them understand the side effects of their treatments. Of the total 1323, 757 (57%) responded the site was moderately or very helpful in this regard (Table 3). Also, 559 patients (42%) agreed that the site had helped them find another patient who had helped them understand what it was like to take a specific treatment for their condition. More patients found the site helpful with decisions to start a medication (n = 496, 37%) than with decisions relating to changing a medication (n = 359, 27%), changing a dosage (n = 336, 25%), or stopping a medication (n = 290, 22%).

**Table 3:** Reported utility of the site for medication-related issues and symptom management

How helpful has PLM been in…? (Category and Question)		Very Helpful	Moderately Helpful	A Little Helpful	Not at all Helpful	N/A, Never Tried to use for This
		n (%)	n (%)	n (%)	n (%)	n (%)
**Treatments**						
	Understanding possible side effects of a medication for your condition	472 (36%)	285 (22%)	167 (13%)	74 (6%)	324 (25%)
	Locating another person who helped you understand what it is like to take a specific medication for your condition	385 (29%)	174 (13%)	164 (12%)	101 (8%)	498 (38%)
	Decisions about whether to start using a medication for your condition	266 (20%)	230 (17%)	127 (10%)	131 (10%)	568 (43%)
	Decisions to change the medication you use to treat your condition	166 (13%)	193 (14%)	131 (10%)	149 (11%)	683 (52%)
	Decisions about whether to change the dose of a medication for your condition	144 (11%)	192 (15%)	127 (10%)	159 (12%)	700 (53%)
	Decisions to stop using a medication for your condition	127 (10%)	163 (12%)	128 (10%)	161 (12%)	743 (56%)
**Symptoms**						
	How helpful has PLM been in learning about a symptom or symptoms you experienced?	639 (48%)	313 (24%)	178 (14%)	55 (4%)	136 (10%)
	How helpful has recording your symptoms been to help you manage your condition?	476 (36%)	309 (23%)	264 (20%)	108 (8%)	164 (12%)
	How helpful have symptom ratings on your profile been in understanding how your treatments are working?	399 (30%)	334 (25%)	279 (21%)	110 (8%)	199 (15%)

### Symptom Management

In a similar fashion to the tools available for monitoring treatments and learning from aggregated data, patients can also benefit from symptom tools. Patients were asked to rate their symptoms on a scale of “none,” “mild,” “moderate,” or “severe.” Each community had about 10 “primary symptoms” that were asked of all patients with that condition; users could also opt to add their own “secondary symptoms,” from which duplicates were removed and errors were corrected. Aggregated reports showed which treatments were being used to treat each symptom. Table 3 shows the benefits gained from symptom tools. Relative to the treatment tools, the symptom tools were more widely used by patients; patients found the site particularly helpful in learning about a symptom they had experienced; 952 of the total 1323 members (72%) reported the site was moderately or very helpful. The majority of members found the site helpful to manage symptoms (n = 785, 59%) and understand how their treatments were working (n = 733, 55%).

#### Association Between Site Use and Treatment/Symptom Management Benefits

To test the hypothesis that the degree of site use (engagement) is associated with benefit, we analyzed the web-logs of participants in the survey to determine how many of the following activities they had engaged in on the site at least once: look at another patient’s profile, open the private message inbox, post in the forum, and start a new topic in the forum. These four activities were turned into a binary 0 or 1 response option and summed to produce an “engagement” score that ranged from 0 to 4. In order to maximize the sample, partially completed surveys were included in this section of the analysis. In Table 4 results from one of the items from Table 3 (“How helpful has PatientsLikeMe been in locating another person who helped you understand what it is like to take a specific medication for your condition”) are compared against the engagement score. There are significant differences between engagement scores for utility (χ^2^_8_=109.4, *P* < .001), with patients who use the site more often finding more benefit.

**Table 4:** Relationship between number of site activities and treatment benefit in locating another patient with experience of taking a specific medication

Engagement Score	Very Helpful	A Little or Moderately Helpful	Didn’t Try or Not Helpful
	n (%)	n (%)	n (%)
0 activities	49 (16%)	78 (25%)	190 (60%)
1 activity	49 (20%)	60 (24%)	140 (56%)
2 activities	103 (27%)	100 (26%)	179 (47%)
3 activities	64 (31%)	61 (29%)	84 (40%)
4 activities	153 (46%)	87 (26%)	92 (28%)

#### Medical Management

As a means of communicating with their health care professional (HCP), a patient could print out their patient profile as a “doctor visit sheet” (DVS) that contained a summary of their outcomes, treatments, and symptoms. About a third of patients (388 of 1323, 29%) reported using the DVS during health care visits (see Table 5). Furthermore, 42% of patients (n = 562) reported being either “moderately” or “a lot more” involved in treatment decisions because of what they learned on PatientsLikeMe. A number of questions were asked about the HCP team’s view of the patient’s use of PatientsLikeMe and 66% (n = 871) reported their HCPs were supportive of their use of PatientsLikeMe. Respondents agreed that the site improved their ability to cope with problems in their life (n = 921, 70% agreed or strongly agreed), that as a result of meeting other patients through the site they felt less self-conscious about their condition (n = 895, 68%), that the site made them feel more in control of their condition (n = 949, 72%) and that it enhanced their quality of life (n = 823, 62%). The majority of respondents, (1,004 or 76%), agreed with the statement: “PatientsLikeMe has helped me understand my own prognosis.”

Following anecdotal reports from the forums, we asked patients across our communities to respond to the statement: “As a result of using PatientsLikeMe, I have changed my physician.” There were significant between-groupdifferences (χ^2^ 15 = 42.9, *P* < .001); overall, 12% (n = 151 of 1320) of patients agreed or strongly agreed, while 88% (n = 1169) of patients disagreed or strongly disagreed. The group that reported the highest rate of changing physician due to PatientsLikeMe use was the fibromyalgia group in which 32 of 150 respondents (21%) strongly agreed or agreed with the statement, followed by the MS group (n = 51 of 344, 15%), the Parkinson’s disease group (n = 26 of 287, 9%), the HIV group (n = 15 of 177, 8%), and the mood disorders group (n = 14 of 144, 10%); the ALS group had the lowest rate (n = 13 of 218, 6%).

**Table 5:** Reported utility of the site for communicating with their health care provider (HCP) using the doctor visit sheet (DVS)

	A Lot	Moderate Amount	A Little	Not at All	Not Applicable, Never Tried to Use Site for This
	n (%)	n (%)	n (%)	n (%)	n (%)
How much do you use the DVS in visits with your HCP team?	91(7%)	136 (10%)	161 (12%)	516 (39%)	417 (32%)
Compared with before PatientsLikeMe (PLM), how much more involved in treatment decisions are you because of what you learned from PLM?	277(21%)	285(22%)	296 (22%)	200 (15%)	263 (20%)
How much easier is it to communicate with your HCP team because of PLM?	281(21%)	300(23%)	284 (22%)	189 (14%)	267 (20%)

### Condition-specific Benefits

Patients from each community were asked a number of condition-specific questions in addition to the core survey items; data from patients with HIV (n = 177) and mood disorders (n = 141) are presented here. Of respondents in the HIV group, 71% (n = 125) agreed or strongly agreed that they took more of an interest in their lab values (ie, cluster of differentiation 4 [CD4] and viral load) because of the site; 63% (n = 111) agreed they had better understanding of the consequences of taking a “drug holiday”; 41% (n = 72) agreed they had decreased risky behaviors; 29% (n = 51) agreed it had helped them decide to start taking antiretroviral drugs.

In the mood disorders community, 26% (n = 40) of users agreed or strongly agreed that using the site had reduced thoughts about self harm; 23% (n = 31) agreed they had decided to start therapy or counseling after interacting with others on the site; and 22% (n = 34) agreed they needed less inpatient care as a result of using PatientsLikeMe.

### Diagnosis Status and Change in Confidence About the Diagnosis

One concern about online community participation is that patients may self-diagnose and do so incorrectly. Respondents were asked: “Did you have a diagnosis when you first became a PatientsLikeMe member?” The vast majority of respondents (1249 of 1323, 94%) stated they already had a diagnosis at the time of joining. Of the patients who still did not have a diagnosis (n = 72), 12 (17%) stated they were awaiting test results; 2 (3%) had not yet consulted a physician; and 1 (1%) was awaiting a second opinion. There were significant between-group differences for diagnostic confidence (χ^2^_20_ = 90.37, *P* < .001). In total, 90% of HIV patients (n = 158 of 176) and 88% of MS users (n = 295 of 336) were “very” or “extremely” confident in their diagnosis, followed by 85% of ALS users (n = 179 of 210), 81% of fibromyalgia patients (n = 117 of 145), 80% of Parkinson’s disease users (n = 230 of 286), and 67% of mood users (n = 93 of 139). Patients were asked whether use of the site had changed their level of confidence that they had the correct diagnosis. Most users (n = 824 of 1292, 64%) reported no change in their diagnostic confidence; 35% (n = 456 of 1292) reported that use of the site had improved their confidence; only 1% of respondents (n = 12 of 1292) reported a decrease in confidence.

### Sharing Medical Data

Respondents were asked, “When you first joined PatientsLikeMe, how comfortable were you with sharing your health information with other users of the site?” and then, “How comfortable are you today with sharing your health information with other users of the site?” At joining, 1090 of 1294 (84%) of respondents were “comfortable” or “very comfortable,” rising to 94% (n = 1212 of 1294) at the time of survey. Between joining and the time of the survey, most respondents remained comfortable sharing health information or became more comfortable, with 69% (n = 889 of 1294) reporting no difference, 27% (n = 354 of 1294) being more positive about sharing, and only 4% (n = 51 of 1294) more negative.

Looking at only those patients who reported being “uncomfortable” or “very uncomfortable” on first joining (16% of users, n = 204), 72% (n = 146 of 204) became more favorable to sharing their health data online since joining; 28% (n = 58 of 204) reported no change; none reported being less comfortable. Comfort levels differed significantly by community on joining (χ^2^ _15_ = 47.52, *P* < .001); initial discomfort (“uncomfortable” or “very uncomfortable”) was highest in patients with mood disorders (n = 26 of 139, 19%) and Parkinson’s disease (n = 52, 18%); lowest levels were in the HIV group (n = 24, 14%) and ALS group (n = 28 of 286, 13%). There was no significant between-group difference in changes in comfort level between joining and time of survey (χ^2^ 30 = 34.94, *P* = .25). At the time of survey, Parkinson’s (n = 22 of 286, 8%) and Fibromyalgia patients (n = 12 of 145, 8%) were the groups with the greatest level of discomfort with sharing, followed by patients with mood disorders (n = 10 of 139, 7%), MS (n = 20 of 338, 6%), HIV (n = 9 of 176, 5%) and ALS (n = 9 of 210, 4%).

## Discussion

### Principal Results

PatientsLikeMe is an ongoing experiment in which patients can gain from sharing and discussing health information online. The site design extends the functionality of traditional qualitative online patient communities to encompass quantitative patient-reported data. Our primary hypothesis was that increased use of the PatientsLikeMe system would lead to greater perceived benefits to patients; our survey suggests that perceived benefits were widespread. Respondents reported learning about symptoms they had been experiencing, improved understanding of how their treatments were working, feeling more involved in treatment decisions, and communicating better with members of their HCP team. As is consistent with findings about members of other online communities [18], members of the PatientsLikeMe site self-reported that the site improved the psychological experience of living with their conditions. Respondents confirmed higher levels of quality of life and perceived control over their condition due to their participation.

Our findings are in line with findings from the recent national consumer survey on health information technology [31], which found that although only about 7% of respondents had ever used a personal health record, those that did reported greater empowerment in managing their health. Reported benefits included feeling that they knew more about their health (56%), feeling like they knew more about the care they were being given by their doctor (52%), and feeling able to ask their doctor questions they would not have asked otherwise (40%).

### Treatment Decisions

Research in the general adult population has suggested that many Internet users (up to 60%) use online information to affect a decision on how to treat an illness or condition, and that an increasing number of Internet users (currently 45%) specifically look for information about prescription or over-the-counter drugs [10]. From our sample of chronic and seriously ill patients, the clearest treatment benefits seem to be around improved understanding of side effects.

Exposure to PatientsLikeMe may represent an opportunity to improve upon the deluge of information provided in drug package inserts [32] and the lack of personal experience or practical advice that a HCP can have with the personal impact of side effects. Results from the survey suggest that members of the site were more likely to research a treatment ahead of time or understand possible effects of taking the drug, rather than making changes to an existing treatment regimen. Some patients (about 1 in 5) did use information from the site to help decide to stop a medication; further research could establish what types of medications these were.

### Symptom Management

A higher proportion of benefits were identified for improving information about patients’ symptoms than for treatments; 59% of users said recording their symptoms on the site had been “helpful” or “very helpful” in managing their condition. Future work could examine whether prospective recording of symptoms is useful in clinical encounters from the provider’s point of view.

#### Association Between Site Use and Treatment Management Benefits

We used our web-logs to analyze the relationship between use of site features and perceived benefit; greater engagement was associated with greater perceived benefit. Specifically, we reported that patients who engaged with the site more were more likely to have identified someone who they could communicate with about a specific treatment. Further analysis (not reported here) suggested that patients who found another patient like them to discuss treatments reported the site was more useful in helping to make treatment decisions such as starting a medication, changing a dose, stopping a medication, or understanding side effects. However, further research will be needed to elucidate the causal chain: is it that engaged users find more benefit, or that patients who have benefitted from the site come back to use more of its features?

#### Medical Management

Two thirds of patients felt that their HCP team approved of using PatientsLikeMe; further follow-up studies could explore the specific benefits experienced by HCPs. It is our hope that office visits can be more focused and effective because use of the doctor visit sheet takes some of the guesswork out of the interaction, particularly with respect to symptom management. Furthermore, constant access to “expert patients” to ask low-level questions means patients can make more effective use of their HCP’s time. Finally, even being a passive recipient of medical knowledge may have a positive effect on condition-specific health literacy, acknowledged to be a significant predictor of outcomes [33].

Varying by disease, between 6% and 21% of site members changed their physician as a result of using the site. This proportion was highest in the fibromyalgia group, a condition where patients anecdotally struggle to find a clinician who will treat them as a medical patient rather than a psychiatric case, but was also high in the MS group. We speculated that the PatientsLikeMe site offers patients novel opportunities to learn about the medical care and experiences of health interactions that other patients like them experience, and a forum to exchange information about “good” doctors in their local area. Again, anecdotally, there is also a push from patients to encourage newly diagnosed members to seek the care of a specialist in their condition. This finding contrasts with the consumers and health information technology (CHIT) study, which found that users of personal health records said they had become less likely to switch doctors as a result of using a personal health record [31]. Further research is needed to examine this interesting finding.

### Condition-specific Benefits

Condition-specific responses provide examples of how the site has helped improve outcomes. For example, medication adherence is a significant problem among patients with HIV for a variety of reasons [34]. The tools provided by the PatientsLikeMe website in the HIV community have helped patients understand their CD4 and viral load test results and the risks inherent in taking a “treatment holiday.” Although the level of participation by members of any given community varies significantly, the HIV community currently stands at over 2700 patients; improved medication compliance among even a fraction of these users could have substantial benefits for those patients, the wider health system, and society as a whole. Encouragingly, 22% of mood disorders group said they needed less inpatient care as a result of using the site, and 26% agreed they think less about self-harm. These findings were not inevitable; there has been some evidence of Internet-related increases in depressive symptoms among patients who are highly engaged in online discussions, speculated to result from rumination or overattention to health problems [35]. Our own site emphasizes data-driven decision making over social functions such as the forum, and future work could look at the effects of participation in the forum as a potential exacerbating factor for some individuals with mood disorders.

### Diagnosis Status

In any online community for patients, it is important to establish what proportion are diagnosed patients as opposed to people who are concerned that a symptom (eg, a muscle twitch) might indicate a serious disease such as ALS. The use of the Internet to self-diagnose has been dubbed “cyberchondria,” and in our experience the presence of undiagnosed patients in a community can be a source of irritation to patients coping with a serious medical condition. We were pleased to find that 94% of respondents had a diagnosis at the time of joining the site as this helps to deliver on the value proposition that prospective users will find a “patient like me” within their community; most e-patients (66%) are searching for advice for a specific medical problem rather than a symptom or undiagnosed condition [10].

### Sharing Medical Data

In the current study we sought to understand patient attitudes to sharing their health data online. Patients who opt to join the site are, by and large, already comfortable with the notion of sharing their health data when they join. However, patients may have fears about potential risks of sharing their personal health data, such as discrimination by employers, insurance companies, or friends and families, particularly in stigmatized illness such as HIV or mood disorders. There are also a variety of real (and imagined) potential “data intruders” on the Internet with motivations ranging from personal research, genealogy, ancestry, forensic purposes or use in marketing, insurance, or employment decisions [36]. Among respondents in the CHIT study 75% who were not using a PHR reported worry about the privacy of their information as one of the most important reasons for not using PHRs, as compared with 51% of respondents who had concerns about cost, 38% who were concerned with how much time it would take, and 26% who did not like computers or the Internet [31]. Although a realistic possibility to be defended against, only 3% of e-patients have reported that they or someone they know has ever been harmed by following medical advice or health information found on the Internet [10]; this figure might compare favorably with advice from health care providers.

Those patients with the most serious illnesses were most comfortable with sharing, suggesting that patients are making risk/benefit analyses about sharing their health data and taking prognosis into account. Given the high initial rate of comfort with sharing, this is likely to reflect a ceiling effect. Although this finding may suggest that sites such as PatientsLikeMe may widen the “digital divide” between those who choose to share and those who do not, it is worth emphasizing that sharing of data was not a prerequisite to registering on the site or benefitting from the data contributed by others. Even without registering (which required only a valid email address), some 20% of patients opted to share their data publicly with anyone on the Internet, without the need to register their personal details on the site (members were explicitly warned that their results could be indexed by search engines if they set their profile to public). All aggregated treatment and symptom data were also shared publicly as reports. Therefore while the greatest benefits were for those that opted to share, anyone may have gained from the site’s database. However, it remains to be seen what the long-term consequences might be of sharing personal health data with the public online.

### Limitations

There are a number of limitations inherent in the study design that should be noted. First as a single survey we were not able to compare attitudes or changes in outcomes with any other population or measure change as it occurred over time. Second, the attitudes assessed in this study may not generalize to a broader population. The patients that opted to join the site may already have been highly involved in their care and comfortable with sharing health information. Therefore it was possible that the site was mostly benefitting patients who were among the most empowered of users anyway. Thirdly, there is likely a response bias whereby patients who have benefitted from the site would be most willing to spend their time completing a survey in their spare time; therefore all positive findings should be interpreted conservatively. With respect to responder bias, the age difference between respondents and nonrespondents is typical of surveys of other populations, that is, younger members of the population are less likely to respond. The differences are relatively small and so in our view do not compromise the sample’s ability to represent our online community.

Fourth, as with any self-report study, it is not possible to say with certainty that all “patients” were correctly diagnosed with their reported condition, nor whether the survey was completed in the presence of a caregiver. Validation studies using clinical studies are the gold standard in replicating self-report registries [37], but such activities are resource-intensive. Finally there are bound to be social biases in the experimenters (who have built the site and so were hoping for a positive benefit) and in the participants (who, if they have benefitted, would have wanted to express their appreciation). That said, the survey had a large sample size and was subject to many of the same biases that exist in any service evaluation.

### Conclusions

Our survey found that a substantial proportion of members of PatientsLikeMe experience benefits from participating in the community. Individually, some system features are relatively underused by patients; we might speculate therefore that much of the benefit identified here comes from peer-peer interaction to aid decision-making [10] as much as from structured data aggregation. Patients reported making more informed treatment decisions as a result of using the site, particularly around managing side effects. Members felt that they were managing their symptoms better and were better able to communicate with peers experiencing the same problems. Patients who used more of the site’s features reported greater benefit, but further research is needed to elucidate the mechanism.

A substantial minority of patients (about a third) reported using data from their profile data in visits with their HCPs. Future work could survey HCPs about the utility of the information collected and displayed in order to increase this number. Collaboration with a clinical service in one of the disease areas covered by PatientsLikeMe could examine the impact of data sharing on clinic visits. Approximately 12% of patients reported having changed their physicians; this may reflect a groundswell of dissatisfaction among patients with chronic conditions and represents another important area for follow-up. Some of the condition-specific benefits are extremely important in improving patient outcomes; quality of life is notoriously difficult to improve in chronic conditions and should be investigated systematically and over a longer time period. The condition-specific benefits identified in the mood disorders and HIV groups hold great potential to improve outcomes for patients with those conditions. Future work should ensure third-party validation and replication of these findings, including gathering data from patients who chose not to join the site.

## Multimedia appendix 1

User survey questionnaire

## Multimedia appendix 2

Open text response comments

## Abbreviations

ALS: amyotrophic lateral sclerosis
ANOVA: analysis of variance
BUILD: building user involvement in motor neurone disease
CD4: cluster of differentiation 4
CHIT: center for health information technology
DVS: doctor visit sheet
HCP: health care professional
HIV: human immunodeficiency virus
IRB: institutional review board
IP: Internet protocol
IRB: institutional review board
MS: multiple sclerosis
PD: Parkinson’s disease
PHR: personal health record
PLM: PatientsLikeMe
