# Supplementary material for: Sharing Health Data for Better Outcomes on PatientsLikeMe
Source: J Med Internet Res. 2010 Jun 14;12(2):e19. doi: 10.2196/jmir.1549 (PMC2956230; doi:10.2196/jmir.1549)
Supplement: Supplementary file 1 [file jmir_v12i2e19_app1.pdf]

PatientsLikeMe Survey Outline
MSers - Has PatientsLikeMe had an impact for you?(2)
Dear PatientsLikeMe User,

Please take a few minutes to tell us about your use of the site - which parts of the site you've found most useful, whether you think PatientsLikeMe has had an impact on your outcomes, and any feedback you may have for us about the site.

You are under no obligation to take part and your answers will be treated in confidence. Some of the aggregated results may be published in scientific journals, on our blog, or in marketing activities, but you will never be personally identified and will remain anonymous. The survey should take you about 10 minutes to complete, although your experience may vary depending on your answers .If you have any questions or suggestions please do not hesitate to get in touch.

Yours sincerely,

Paul Wicks, Mike Massagli, and the PatientsLikeMe R&D Team

Section 1. **Diagnosis**

1. **Did you have a diagnosis when you first became a PatientsLikeMe member?**
   Key: HAVEDX
   Format: Multiple-choice
   - Yes
   - No

Section 2. **Branch - Dx'd now?**

Show if: #HAVEDX IS 'No'

1. **Do you now have a diagnosis?**
   Key: NOWHAVEDX
   Format: Multiple-choice
   - Yes
   - No

Section 3. **Branch - Why no dx?**

Show if: #NOWHAVEDX IS 'No'

1. **What is the reason you do not have a diagnosis?**
   Key: REASONNODX
   Format: Multiple-choice
   - You have not consulted physician or specialist
   - You are still waiting for test results
   - You are waiting for referral for a second opinion
   - Another reason

Section 4. **Branch - Other reason describe**

Show if: #REASONNODX IS 'Another reason'

1. **Use this space to tell us why you do not have a diagnosis.**
   Key: *none*
   Format: Free-form text

Section 5. **Obtaining your diagnosis**

Show if: #NOWHAVEDX IS 'Yes'

1. **Was information you found on PatientsLikeMe or received from other PatientsLikeMe users helpful in obtaining your diagnosis?**
   Key: INFODX
   Format: Multiple-choice
   - Yes
   - No

Section 6. **Confidence in diagnosis**

Show if: #HAVEDX IS 'Yes' OR #INFODX IS 'Yes' OR #INFODX IS 'No'

1. **How confident are you that you have received the correct diagnosis?**
   Key: *none*
   Format: Multiple-choice
   - Extremely confident
   - Very confident
   - Moderately confident
   - A little confident
   - Not confident at all

2. **When you *first joined* PatientsLikeMe, how comfortable were you with sharing your health information with other users of the site?**
   Key: *none*
   Format: Multiple-choice
   - Very comfortable
   - Comfortable
   - Uncomfortable
   - Very uncomfortable

3. **How comfortable are you *today* with sharing your health information with other users of the site?**
   Key: *none*
   Format: Multiple-choice
   - Very comfortable
   - Comfortable
   - Uncomfortable
   - Very uncomfortable

4. **Has using the PatientsLikeMe site:**
   Key: *none*
   Format: Multiple-choice
   - Increased your confidence in your diagnosis
   - Decreased your confidence in your diagnosis
   - Not changed your confidence in your diagnosis

5. **(Not including today) during the past 4 weeks, about how often have you logged in to use PatientsLikeMe?**
   Key: LOGIN
   Format: Multiple-choice
   - Daily
   - A few times per week
   - About once per week

- Less than once per week
- Have not logged in during the past 4 weeks

Section 7. **Branch - Why haven't you logged in?**

Show if: #LOGIN IS 'Have not logged in during the past 4 weeks'

1. **Please use the space provided and tell us why you have not logged in during the past 4 weeks.**
   Key: *none*
   Format: Free-form text

Section 8. **Treatment Decisions**
In this section, please rate how helpful PatientsLikeMe has been in...

1. **decisions to change the medication you use to treat your condition**
   Key: *none*
   Format: Multiple-choice
   - Not at all helpful
   - A little helpful
   - Moderately helpful
   - Very helpful
   - Does not apply - never tried to use for this

2. **decisions about whether to change the dose of a medication for your condition**
   Key: *none*
   Format: Multiple-choice
   - Not at all helpful
   - A little helpful
   - Moderately helpful
   - Very helpful
   - Does not apply - never tried to use for this

3. **decisions about whether to start using a medication for your condition**
   Key: *none*
   Format: Multiple-choice
   - Not at all helpful
   - A little helpful
   - Moderately helpful
   - Very helpful
   - Does not apply - never tried to use for this

4. **understanding possible side effects of a medication you have taken for your condition**
   Key: *none*
   Format: Multiple-choice
   - Not at all helpful
   - A little helpful
   - Moderately helpful
   - Very helpful
   - Does not apply - never tried to use for this

5. **locating another person who helped you understand what it is like to take a specific medication for your condition**
   Key: *none*
   Format: Multiple-choice

- Not at all helpful
- A little helpful
- Moderately helpful
- Very helpful
- Does not apply - never tried to use for this

6. **decisions to stop using a medication for your condition**
   Key: *none*
   Format: Multiple-choice
   - Not at all helpful
   - A little helpful
   - Moderately helpful
   - Very helpful
   - Does not apply - never tried to use for this

Section 9. **Treatment - II**

1. **PatientsLikeMe has helped me take my medications more regularly**
   Key: *none*
   Format: Multiple-choice
   - Strongly disagree
   - Disagree
   - Agree
   - Strongly agree

2. **As a result of using the site I have avoided the wrong treatment**
   Key: *none*
   Format: Multiple-choice
   - Strongly disagree
   - Disagree
   - Agree
   - Strongly agree

3. **PatientsLikeMe has helped me avoid unnecessary treatment**
   Key: *none*
   Format: Multiple-choice
   - Strongly disagree
   - Disagree
   - Agree
   - Strongly agree

4. **PatientsLikeMe has helped me find ways to reduce treatment side-effects**
   Key: *none*
   Format: Multiple-choice
   - Strongly disagree
   - Disagree
   - Agree
   - Strongly agree

Section 10. **Symptoms**
In this section, please rate the following statements about the PatientsLikeMe Symptom tools

1. **How much does recording your symptoms help you manage your condition**
   Key: *none*
   Format: Multiple-choice

- Not at all helpful
- A little helpful
- Moderately helpful
- Very helpful
- Does not apply - never tried to use for this

2. **How helpful have symptom ratings on your profile been in understanding how your treatments are working**
   Key: *none*
   Format: Multiple-choice
   - Not at all helpful
   - A little helpful
   - Moderately helpful
   - Very helpful
   - Does not apply - never tried to use for this

3. **How helpful has PatientsLikeMe been in learning about a symptom or symptoms you experienced**
   Key: *none*
   Format: Multiple-choice
   - Not at all helpful
   - A little helpful
   - Moderately helpful
   - Very helpful
   - Does not apply - never tried to use for this

Section 11. **Symptoms - II**

1. **PatientsLikeMe has helped me locate another person who has the same symptoms that I am experiencing**
   Key: *none*
   Format: Multiple-choice
   - Strongly disagree
   - Disagree
   - Agree
   - Strongly agree

2. **I have received better care from my healthcare provider by recording symptoms on my profile**
   Key: *none*
   Format: Multiple-choice
   - Strongly disagree
   - Disagree
   - Agree
   - Strongly agree

Section 12. **The Forum**
In this section, please rate the following statements about the PatientsLikeMe Forum.

1. **How much have you used information from the Forum during regular visits with your healthcare team**
   Key: *none*
   Format: Multiple-choice
   - Not at all
   - A little

- A moderate amount
- A lot
- Does not apply - never tried to use for this

2. **How much have you used information from the Forum to learn about experimental treatments that may help you personally**
   Key: *none*
   Format: Multiple-choice
   - Not at all
   - A little
   - A moderate amount
   - A lot
   - Does not apply - never tried to use for this

Section 13. **The Forum - II**

1. **I feel more comfortable asking for information on sensitive topics such as sex, sexuality, religion, or spirituality on the PatientsLikeMe forum than other places such as other internet forums or real-world support groups**
   Key: *none*
   Format: Multiple-choice
   - Strongly disagree
   - Disagree
   - Agree
   - Strongly agree

2. **I think the Forum is a good place for social support**
   Key: *none*
   Format: Multiple-choice
   - Strongly disagree
   - Disagree
   - Agree
   - Strongly agree

3. **I think the Forum is a good place for sharing medical information**
   Key: *none*
   Format: Multiple-choice
   - Strongly disagree
   - Disagree
   - Agree
   - Strongly agree

4. **I think the Forum is a good place for learning from others**
   Key: *none*
   Format: Multiple-choice
   - Strongly disagree
   - Disagree
   - Agree
   - Strongly agree

Section 14. **Impact on your relationship with your healthcare providers (9/14)**
In this section, please rate the impact of your use of PatientsLikeMe on your relationship with your healthcare providers.

1. **How much do you use the Doctor Visit Sheet during regular visits with members of your**

healthcare team
Key: *none*
Format: Multiple-choice

- Not at all
- A little
- A moderate amount
- A lot
- Does not apply - never tried to use for this

2. **Compared with before you used PatientsLikeMe, how much more involved in decisions about your treatment are you because of what you have learned from PatientsLikeMe**
   Key: *none*
   Format: Multiple-choice
   - Not at all
   - A little
   - A moderate amount
   - A lot
   - Does not apply - never tried to use for this

3. **How much easier is it to communicate with your healthcare team because you use information from PatientsLikeMe**
   Key: *none*
   Format: Multiple-choice
   - Not at all
   - A little
   - A moderate amount
   - A lot
   - Does not apply - never tried to use for this

Section 15. **HCP - II**

1. **I have problems with coordination of my care among different members of my health care team**
   Key: *none*
   Format: Multiple-choice
   - Strongly disagree
   - Disagree
   - Agree
   - Strongly agree

2. **Using information from PatientsLikeMe has helped my healthcare team give me better care for my condition**
   Key: *none*
   Format: Multiple-choice
   - Strongly disagree
   - Disagree
   - Agree
   - Strongly agree

3. **Making decisions about my treatment with my health care team is easier because of the information I get from PatientsLikeMe**
   Key: *none*
   Format: Multiple-choice
   - Strongly disagree
   - Disagree

- Agree
- Strongly agree

4. **Using information from PatientsLikeMe has helped my healthcare team understand how my treatment is working**
   Key: *none*
   Format: Multiple-choice
   - Strongly disagree
   - Disagree
   - Agree
   - Strongly agree

5. **As a result of using PatientsLikeMe I have changed my physician**
   Key: *none*
   Format: Multiple-choice
   - Strongly disagree
   - Disagree
   - Agree
   - Strongly agree

6. **My healthcare team is supportive of my use of PatientsLikeMe**
   Key: *none*
   Format: Multiple-choice
   - Strongly disagree
   - Disagree
   - Agree
   - Strongly agree

Section 16. **What is the overall impact of your use of PatientsLikeMe? (10/14)**
In this section, please indicate how much you agree or disagree with the following statements about the impact of your use of PatientsLikeMe overall.

1. **I am more informed about my condition because of PatientsLikeMe**
   Key: *none*
   Format: Multiple-choice
   - Strongly disagree
   - Disagree
   - Agree
   - Strongly agree

2. **Using PatientsLikeMe is helping me cope better with problems in my life**
   Key: *none*
   Format: Multiple-choice
   - Strongly disagree
   - Disagree
   - Agree
   - Strongly agree

3. **As a result of using the site I have learned how to get better care**
   Key: *none*
   Format: Multiple-choice
   - Strongly disagree
   - Disagree
   - Agree
   - Strongly agree

4. **As a result of using the site, my overall quality of life is better**
   Key: *none*
   Format: Multiple-choice
   - Strongly disagree
   - Disagree
   - Agree
   - Strongly agree

5. **PatientsLikeMe has helped me understand my own prognosis**
   Key: *none*
   Format: Multiple-choice
   - Strongly disagree
   - Disagree
   - Agree
   - Strongly agree

6. **As a result of meeting other patients through the site I feel less self-conscious about my illness**
   Key: *none*
   Format: Multiple-choice
   - Strongly disagree
   - Disagree
   - Agree
   - Strongly agree

7. **As a result of using the site I have more hope that a cure will be found for this disease**
   Key: *none*
   Format: Multiple-choice
   - Strongly disagree
   - Disagree
   - Agree
   - Strongly agree

8. **As a result of using the site, I have less stress or worry about my condition**
   Key: *none*
   Format: Multiple-choice
   - Strongly disagree
   - Disagree
   - Agree
   - Strongly agree

9. **I feel that using PatientsLikeMe gives me more and better control over my condition**
   Key: *none*
   Format: Multiple-choice
   - Strongly disagree
   - Disagree
   - Agree
   - Strongly agree

Section 17. **Disease-specific questions - MS**
Please rate your agreement or disagreement with the following statements:

1. **Using PatientsLikeMe has helped me understand if I am having a relapse**
   Key: *none*
   Format: Multiple-choice

- Strongly disagree
- Disagree
- Agree
- Strongly agree
- Does not apply to me

2. **Using PatientsLikeMe has helped me get better care when I am having a relapse**
   Key: *none*
   Format: Multiple-choice
   - Strongly disagree
   - Disagree
   - Agree
   - Strongly agree
   - Does not apply to me

3. **Using PatientsLikeMe had given me a better sense of what to expect when a relapse starts**
   Key: *none*
   Format: Multiple-choice
   - Strongly disagree
   - Disagree
   - Agree
   - Strongly agree
   - Does not apply to me

Section 18. **Some Final Questions About You**
Your answers to the following will help us understand a little about your situation.

1. **About how many times during the past 12 months did you vist a doctor's office for an exam or treatment related to your condition?**
   Key: *none*
   Format: Multiple-choice
   - None
   - 1 time
   - 2 times
   - 3 or 4 times
   - 5 or more times

2. **About how many times during the past 12 months did you stay in a hospital overnight or longer for care or treatment you needed because of your condition?**
   Key: *none*
   Format: Multiple-choice
   - None
   - 1 time
   - 2 times
   - 3 or 4 times
   - 5 or more times

3. **Have you ever stopped a treatment because it made you gain weight?**
   Key: *none*
   Format: Multiple-choice
   - Yes
   - No

4. **Have you ever decided not to take a treatment your doctor recommended because you were afraid it would make you gain weight?**

none

Format: Multiple-choice

- Yes
- No

5. **During the past 12 months, about how much money did you spend out of pocket to pay for prescribed medications to treat your condition?**
   Key: *none*
   Format: Multiple-choice
   - Nothing
   - under $500
   - $501 to $1,000
   - $1,001 to $5,000
   - $5,001 to $10,000
   - more than $10,000

6. **During the past 12 months, about how much money did you spend out of pocket to pay for all other expenses you had to treat your condition?**
   Key: *none*
   Format: Multiple-choice
   - Nothing
   - under $500
   - $501 to $1,000
   - $1,001 to $5,000
   - $5,001 to $10,000
   - more than $10,000

7. **Which of the following comes closest to describing your relationship with the health care provider that provides most of the care for your condition?**
   Key: *none*
   Format: Multiple-choice
   - I feel uninformed about my condition and totally dependent on my provider
   - I am somewhat informed about my condition but defer to my provider for most decisions about my care
   - I am moderately well-informed about my condition and share some responsibility with my provider for most decisions about my care
   - I am very well-informed about my condition and I alone am responsible for nearly all decisions about my care

---

Section 19. **Final comments**

Thank you for taking the time to complete this survey. If there is anything else you would like us to know about how you use the site or how we can make the site more valuable to you, please use the space below. When you are finished, please click the "Submit Survey" button to submit your answers.

1. **What other sites or groups do you belong to on the internet for your condition?**
   Key: *none*
   Format: Free-form text

2. **Please use the space below for your final comments, or if you have a suggestion for one thing you would really like to see change on the site please let us know here:**
   Key: *none*
   Format: Free-form text

Generated May 10, 2010 08:45AM

© 2005-2010 PatientsLikeMe. Information on PatientsLikeMe.com does not constitute medical advice.
